# Drug Dosing During Continuous Renal Replacement Therapy in Critically Ill Patients: A Practical Narrative Review

**DOI:** 10.3390/pharmaceutics18101320

**Published:** 2026-10-18

**Authors:** Mihai Claudiu Pui, Alexandra Elena Lazar, Janos Szederjesi, Oana Elena Branea, Mihaela Butiulca, Vlad-Dimitrie Cehan, Mihaela Straistă, Leonard Azamfirei

**Affiliations:** 1 Doctoral School of Medicine and Pharmacy, George Emil Palade University of Medicine, Pharmacy, Science and Technology of Targu Mures, 540139 Targu Mures, Romania; straista.mihaela.24@stud.umfst.ro; 2 Department of Anesthesiology and Intensive Care Medicine, Emergency County Hospital, 540136 Targu Mures, Romania; alexandra.lazar@umfst.ro (A.E.L.); yangzi37@gmail.com (J.S.); oana.branea@umfst.ro (O.E.B.); mihaela.butiulca@umfst.ro (M.B.); cehan.vlad-dimitrie.24@stud.umfst.ro (V.-D.C.); leonard.azamfirei@umfst.ro (L.A.); 3 Department of Anesthesiology and Intensive Care, George Emil Palade University of Medicine, Pharmacy, Science and Technology of Targu Mures, 540142 Targu Mures, Romania

**Keywords:** continuous renal replacement therapy, renal replacement therapy, drug dosing, pharmacokinetics, pharmacokinetic variability, therapeutic drug monitoring, model-informed precision dosing

## Abstract

Drug dosing during continuous renal replacement therapy (CRRT) is difficult in critically ill patients because drug exposure is influenced by both the patient and the extracorporeal treatment. This narrative review provides a practical overview of drug dosing during CRRT, while intermittent hemodialysis and sustained low-efficiency dialysis are discussed only where they provide relevant contextual or comparative information. It summarizes the main drug-related, patient-related, and treatment-related factors that influence extracorporeal drug clearance and discusses their clinical implications for commonly used drug classes. Loading and maintenance doses should be considered separately: loading doses depend mainly on volume of distribution and the need for early therapeutic exposure, whereas maintenance dosing depends on total clearance, including residual renal, extracorporeal, and non-renal clearance. Therapeutic drug monitoring can help guide dose adjustment when validated targets are available, while model-informed precision dosing may provide additional support in selected patients. The review also proposes an eight-step practical framework based on residual kidney function, the actual delivered CRRT prescription, drug pharmacokinetics and pharmacodynamics, therapeutic monitoring, and repeated reassessment. Future approaches may combine real-time CRRT data with pharmacokinetic models to support more individualized dosing.

## 1. Introduction

Acute kidney injury is common in critically ill patients and is associated with substantial morbidity and mortality. In severe kidney dysfunction, renal replacement therapy (RRT) may be required to maintain solute control, acid–base and electrolyte balance, and fluid management [1]. Continuous renal replacement therapy (CRRT) is frequently used in intensive care, particularly in patients with hemodynamic instability, because it allows gradual and continuous removal of solutes and fluids compared with intermittent modalities [2]. Drug dosing in this setting is particularly challenging. Critical illness, acute kidney injury, residual renal function, hepatic dysfunction, hypoalbuminemia, fluid overload, and systemic inflammation may all alter drug exposure. RRT adds further variability because drug removal depends not only on patient-related factors but also on treatment modality, membrane characteristics, blood flow, dialysate and replacement-fluid flow rates, effluent dose, treatment duration, and actual circuit performance [2,3,4].

Inappropriate dosing during RRT may lead to underexposure and therapeutic failure or, conversely, to drug accumulation and toxicity. This is particularly relevant for antimicrobials and drugs with narrow therapeutic windows, for which adequate and timely exposure is essential. Extracorporeal drug removal is influenced by physicochemical and pharmacokinetic characteristics such as molecular weight, protein binding, volume of distribution, and water solubility. Patient-specific factors and the technical characteristics of RRT further contribute to substantial interindividual variability [3,4,5].

Loading and maintenance doses should therefore be considered separately. In critically ill patients, loading-dose requirements are influenced mainly by the volume of distribution and its changes during acute illness, whereas maintenance dosing depends more closely on total drug clearance, including residual renal, extracorporeal, and non-renal clearance. Consequently, dose adjustment during CRRT and other RRT modalities should not rely solely on estimated glomerular filtration rate or fixed dose-reduction schemes. An individualized approach should integrate drug characteristics, residual kidney function, RRT modality and delivered intensity, patient-specific physiological changes, and therapeutic drug monitoring (TDM) when available, with repeated reassessment as renal function, CRRT settings, filter performance, albumin concentration, fluid balance, and clinical status change [3,4,6,7].

Although intermittent hemodialysis and sustained low-efficiency dialysis are discussed where relevant, the primary focus of this review is continuous renal replacement therapy in critically ill adults. Evidence from intermittent hemodialysis and sustained low-efficiency dialysis is interpreted as contextual or indirect support and is not intended to provide comprehensive modality-specific dosing guidance for these techniques.

This review aims to synthesize clinically relevant pharmacokinetic principles and practical dosing considerations for commonly used ICU drugs during continuous renal replacement therapy, with emphasis on extracorporeal drug clearance, patient-related pharmacokinetic variability, delivered treatment intensity, therapeutic drug monitoring, and individualized dosing strategies.

## 2. Materials and Methods

This narrative review was designed as a structured, non-systematic narrative review to identify and synthesize clinically relevant evidence on drug dosing during continuous renal replacement therapy (CRRT) in critically ill adults. Evidence from intermittent hemodialysis, sustained low-efficiency dialysis, and broader renal replacement therapy literature was considered where it provided relevant contextual, comparative, or indirect pharmacokinetic support. The review was organized around predefined thematic domains: mechanisms of extracorporeal drug clearance, pharmacokinetic determinants of drug removal, technical determinants of CRRT-related clearance, patient-related determinants in critical illness, drug-class-specific dosing implications, therapeutic drug monitoring, and individualized dosing approaches. PubMed was used as the primary bibliographic database, supplemented by targeted Google Scholar searches and screening of relevant reference lists. The search covered literature published from January 2015 to September 2026, while older studies were retained when they provided essential physiological, pharmacokinetic, or methodological background. The initial search was performed before submission and was updated during revision. The literature search was last updated on 10 September 2026, with an additional targeted update performed during final revision to include recent CRRT-specific evidence. Only English-language publications were considered. Evidence was restricted primarily to adult populations, and pediatric-only studies were excluded. Mixed-population guidelines or consensus documents were retained when they contained recommendations directly applicable to critically ill adults. A transparent search log is provided in Supplementary Table S1, including the main PubMed search blocks, predefined search domains, search window, date of last search, and supplementary targeted search approaches. Google Scholar was used as a supplementary targeted source to identify additional clinically relevant articles, guidelines, expert reviews, and pharmacokinetic studies, while reference-list screening was used for citation tracking. These approaches were not used as systematic databases of record and therefore did not generate a formal screening count.

Eligible publications included clinical and pharmacokinetic studies, population pharmacokinetic and pharmacometric studies, systematic and narrative reviews, guidelines, consensus documents, and selected in vitro studies directly relevant to membrane adsorption or extracorporeal drug removal. Studies without direct pharmacokinetic or pharmacological relevance to RRT were excluded. Additional exclusion criteria were non-adult populations without applicability to adult critical care, absence of clinically relevant dosing or clearance information, studies focused on drug classes outside the scope of the review, and publications that duplicated information already addressed by more specific pharmacokinetic studies, guidelines, consensus documents, or comprehensive reviews. Evidence was interpreted hierarchically. Direct drug-specific pharmacokinetic studies in critically ill patients receiving CRRT and validated TDM targets were given priority for dosing-related statements. Guidelines, consensus documents, observational studies, systematic and narrative reviews, in vitro data, evidence from IHD or SLED, simulation studies, healthy-volunteer data, and pharmacokinetic reasoning were used as supportive or indirect evidence where direct CRRT data were limited. Evidence derived from non-CRRT populations, other RRT modalities, in vitro experiments, simulation studies, or general pharmacokinetic principles was interpreted cautiously and identified as indirect or extrapolated where relevant. Statements based on limited, inconsistent, or indirect evidence were presented as practical considerations rather than fixed dosing recommendations. When findings were discrepant, they were interpreted according to study population, CRRT modality, delivered effluent rate, residual kidney function, membrane characteristics, dosing strategy, and availability of TDM. Drug classes were selected according to their relevance to intensive care practice and the potential clinical consequences of altered exposure during RRT. The drugs included in the practical tables were selected as representative clinically relevant examples rather than as an exhaustive list, based on their frequency of use in critically ill patients, the potential importance of CRRT-related changes in exposure, diversity of pharmacokinetic characteristics, and availability of drug-specific evidence. The drugs included in Supplementary Table S2 were selected as illustrative examples among ICU antimicrobial agents for which the cited literature reported both a dosing regimen and the corresponding CRRT modality and flow setting. The table was not intended to be exhaustive, and drugs were not included when the cited sources did not clearly report a regimen together with the relevant CRRT setting. To increase the practical value of the review without presenting author-derived fixed dosing recommendations, a supplementary table was added summarizing published CRRT dosing regimens for selected ICU antimicrobial agents. These regimens were extracted from cited pharmacokinetic studies, reviews, or dosing analyses when the CRRT modality, delivered effluent rate or CRRT dose, dosing regimen, and relevant PK/PD target were reported. Literature identification and initial thematic selection were performed by one author (M.C.P.). The final selection of clinically relevant domains, drug classes, and practical interpretations was reviewed by the author group during manuscript revision. Because this was a structured narrative review rather than a systematic review, duplicate independent screening was not performed. No systematic-review protocol was registered, and no formal risk-of-bias or GRADE assessment, PRISMA flow diagram, or quantitative synthesis was performed. A narrative approach was chosen because the available evidence is heterogeneous across RRT modalities, treatment prescriptions, membrane characteristics, patient populations, pharmacokinetic methods, therapeutic targets, and drug-specific outcomes. The resulting synthesis was used to develop an author-proposed CRRT-focused dosing framework. The steps were generated by grouping recurring determinants identified across the reviewed literature, including residual kidney function, delivered CRRT intensity, drug-specific PK/PD characteristics, loading-dose requirements, maintenance-dose adjustment, TDM, and repeated reassessment. The sequence was ordered to reflect a bedside decision process, moving from patient and CRRT-related determinants to drug-specific exposure targets, dose selection, monitoring, and reassessment. The review is intended to provide a framework for individualized dosing rather than a comprehensive catalogue of fixed drug-specific dosing regimens.

## 3. Principles of Extracorporeal Drug Clearance

Extracorporeal drug clearance during renal replacement therapy is mainly determined by three mechanisms: diffusion, convection, and adsorption. Their relative contribution depends on the RRT modality, membrane characteristics, and the properties of the drug.

Diffusion is the main mechanism of solute transfer during intermittent hemodialysis and diffusive CRRT modalities. It depends on the concentration gradient between blood and dialysate across a semipermeable membrane. Small, water-soluble drugs with limited protein binding are generally more susceptible to diffusive removal. Increasing dialysate flow may enhance clearance, although the effect also depends on blood flow, membrane characteristics, and the unbound fraction of the drug [2,8].

Convection is the dominant mechanism during hemofiltration and contributes during hemodiafiltration. Plasma water is driven across the membrane, carrying dissolved molecules by solvent drag. Convective clearance depends mainly on ultrafiltration flow, membrane permeability, sieving coefficient, and the unbound drug fraction. Compared with diffusion, convection may contribute more to the removal of larger molecules that can cross the membrane [2,8].

Adsorption results from binding of molecules to the membrane or cartridge. Its importance varies between drugs and filter materials and may be greatest early after filter initiation, before binding sites become saturated. In vitro studies have demonstrated variable antimicrobial adsorption across CRRT filters, suggesting that membrane type and filter age may contribute to changes in measured drug exposure [9]. The overall extracorporeal clearance of a drug therefore reflects the interaction between these mechanisms and the actual CRRT delivered. During CRRT, interruptions, circuit clotting, and filter changes may cause delivered clearance to differ from the prescribed treatment [2,4,8].

## 4. Pharmacokinetic Determinants of Drug Removal

Drug removal during RRT depends on both the extracorporeal treatment and the pharmacokinetic characteristics of the drug. The main drug-related determinants are molecular weight, protein binding, volume of distribution, and hydrophilicity, while residual renal and non-renal clearance determine the contribution of RRT to total drug clearance [3,4,5,6].

Molecular weight influences membrane passage. Small molecules are generally more readily removed by diffusion and convection, particularly when they are water-soluble and predominantly unbound. Larger molecules cross standard dialysis membranes less efficiently, although membrane permeability may modify this relationship. Molecular weight alone is therefore insufficient to predict extracorporeal removal [2,4,8].

Protein binding determines the fraction of drug directly available for extracorporeal removal. The unbound fraction is freely available for diffusion or filtration across the membrane, whereas highly protein-bound drugs are generally less susceptible to extracorporeal clearance. In critically ill patients, hypoalbuminemia may increase the unbound fraction of highly protein-bound drugs, potentially increasing the fraction available for extracorporeal removal and complicating interpretation of total plasma concentrations. For selected highly protein-bound drugs with narrow therapeutic windows, total concentrations may not reflect active unbound exposure when albumin concentration or protein binding is altered; measurement of unbound concentrations may therefore provide additional information when available [3,4,5,6].

Volume of distribution is another major determinant of extracorporeal removal. Drugs with a low Vd remain mainly within the intravascular and extracellular compartments and are generally more accessible to RRT. In contrast, drugs with a large Vd distribute extensively into tissues, leaving a smaller circulating fraction available for extracorporeal removal [3,4,5,6].

Hydrophilicity and lipophilicity further influence drug distribution and extracorporeal removal. Hydrophilic drugs tend to remain within plasma and extracellular fluid and are generally more susceptible to removal by dialysis or hemofiltration. Lipophilic drugs distribute more extensively into tissues and adipose compartments and frequently have larger volumes of distribution, making extracorporeal removal less clinically important or less predictable. This distinction is particularly relevant for many antimicrobials, which are hydrophilic and potentially dialyzable, compared with several sedatives and opioids, for which tissue distribution may limit extracorporeal removal [3,4,10].

Residual renal function and non-renal clearance must be considered together with extracorporeal clearance. Total drug clearance during RRT reflects the combined contribution of residual renal clearance, extracorporeal clearance, and non-renal elimination, including hepatic metabolism and biliary excretion. Residual kidney function may remain clinically relevant even during CRRT; vancomycin provides a well-described example, with residual diuresis contributing to interindividual variability in drug clearance [11]. Conversely, impairment of non-renal elimination may increase the risk of accumulation for drugs that are not efficiently removed by the extracorporeal circuit [4,6,10].

These pharmacokinetic determinants should be interpreted together with patient physiology and the actual CRRT prescription rather than in isolation. Changes in volume of distribution mainly inform loading-dose decisions, whereas total clearance, including residual renal, extracorporeal, and non-renal clearance, primarily informs maintenance dosing. Body size, fluid balance, edema, obesity, and reduced muscle mass may further modify these decisions. In obesity, severe edema, or marked fluid accumulation, the choice between actual, ideal, or adjusted body weight should follow drug-specific pharmacokinetic guidance rather than a uniform rule. In patients with reduced muscle mass, serum creatinine may underestimate renal dysfunction and may be an unreliable marker of residual kidney function; urine output, clinical trajectory, and measured drug concentrations, when available, should therefore be considered alongside creatinine-based estimates [3,4,6,7]. The main pharmacokinetic determinants of extracorporeal drug removal are summarized in Table 1.

## 5. Technical Determinants of CRRT-Related Drug Clearance

The technical prescription of renal replacement therapy is a major determinant of extracorporeal drug clearance. Drug removal is influenced not only by the selected modality but also by treatment intensity, membrane characteristics, treatment duration, and the difference between prescribed and delivered therapy. These variables are particularly important during continuous renal replacement therapy, where extracorporeal clearance may change over time because of treatment interruptions, circuit clotting, filter replacement, or modifications in the delivered prescription [4,12,13]. Because CRRT, intermittent hemodialysis, and SLED differ in treatment duration, instantaneous clearance, off-treatment intervals, and delivered solute removal, evidence from one modality cannot be directly transferred to another without caution [2,3,7,8,13]. Throughout this review, CRRT-specific data are distinguished from evidence derived from intermittent or prolonged intermittent modalities whenever the available literature allows such separation. When modality-specific evidence is limited, dosing implications are presented as extrapolated practical considerations rather than definitive modality-specific recommendations.

RRT modality determines the temporal pattern and mechanism of drug removal. Intermittent hemodialysis provides relatively high instantaneous clearance during a limited treatment period and may require post-dialysis supplementation for highly dialyzable drugs. CRRT provides lower instantaneous but sustained clearance while the circuit remains operational [2,3,13]. Hemofiltration relies predominantly on convection, hemodialysis on diffusion, and hemodiafiltration combines both mechanisms [8,13]. Prolonged intermittent modalities, including sustained low-efficiency dialysis, produce time-dependent clearance characterized by distinct on-treatment and off-treatment pharmacokinetic phases [3,7].

Blood flow determines solute delivery to the extracorporeal circuit and influences the relationship between blood, dialysate, and ultrafiltration flows. However, within commonly used CRRT prescriptions, increasing blood flow does not necessarily result in a proportional increase in drug clearance, particularly when effluent flow is the rate-limiting component. Adequate blood flow remains important for maintaining circuit performance and supporting the prescribed filtration or dialysis intensity, and may be limited by vascular access dysfunction or circuit pressure constraints [8,12,13].

Dialysate flow is a major determinant of diffusive clearance. Higher dialysate flow may increase removal of small, water-soluble, predominantly unbound drugs, although the effect depends on blood flow, membrane characteristics, molecular size, and protein binding [3,8].

Convective clearance is determined primarily by the total ultrafiltration flow through the membrane and the drug’s sieving coefficient rather than by net fluid removal alone [8,12]. Drugs with a high unbound fraction and adequate membrane permeability are more readily removed by convection. When replacement fluid is administered before the filter, predilution lowers the solute concentration entering the membrane and reduces effective clearance compared with an equivalent postdilution prescription. Postdilution avoids this dilutional effect but may increase hemoconcentration and the risk of circuit clotting when filtration fraction is high [8,12,13].

Effluent flow provides a practical bedside surrogate of CRRT intensity and reflects the combined contribution of dialysate and ultrafiltrate flow, with correction for predilution when relevant [12,13]. For many small, hydrophilic drugs, extracorporeal clearance increases as delivered CRRT intensity increases. Maintenance dosing should therefore be reassessed when CRRT intensity changes [3,4,7]. Importantly, prescribed and delivered therapy may differ because of downtime, procedures, alarms, access dysfunction, filter changes, or premature circuit clotting [12,13].

As a simplified bedside estimate, extracorporeal drug clearance during CRRT may be approximated from delivered treatment flows and drug-specific membrane transfer. For predominantly convective clearance, clearance may be estimated as ultrafiltration flow multiplied by the sieving coefficient; for predominantly diffusive clearance, dialysate flow multiplied by the saturation coefficient provides a practical approximation. In hemodiafiltration, both components may contribute, while predilution, protein binding, membrane permeability, adsorption, residual kidney function, and non-renal clearance modify the relationship between effluent flow and actual exposure. For example, a prescribed effluent dose of 25 mL/kg/h in a 70 kg patient corresponds to approximately 1.75 L/h. If treatment downtime reduces effective delivery to 80%, the delivered extracorporeal component would be closer to 1.4 L/h before accounting for the sieving or saturation coefficient and other drug-specific factors. This illustrates why maintenance dosing should be linked to delivered rather than prescribed CRRT intensity and reassessed after interruptions, filter changes, or major prescription changes [3,4,7,12,13].

Membrane characteristics, including permeability, surface area, and adsorption capacity, may further modify drug transfer [3,9]. The clinical relevance of adsorption varies considerably according to drug and membrane type and remains difficult to predict from membrane characteristics alone. In vitro data demonstrate variable antimicrobial adsorption across CRRT filters, supporting consideration of membrane type and circuit age when unexpectedly low or changing drug concentrations are observed [9].

Treatment duration and circuit performance determine the cumulative extracorporeal exposure of a drug. Intermittent therapies produce episodic clearance, whereas CRRT provides continuous clearance only while treatment is effectively running [2,13]. Interruptions reduce delivered extracorporeal clearance and may promote drug accumulation, whereas increases in CRRT intensity may increase removal and contribute to underexposure if maintenance dosing is not reassessed. Changes in circuit function, including recurrent clotting, access dysfunction, or frequent filter replacement, should therefore be considered when interpreting drug exposure [3,4,12].

In clinical practice, delivered rather than prescribed CRRT intensity should be used when interpreting drug exposure and adjusting maintenance dosing [3,4,7]. The main pharmacokinetic determinants of extracorporeal drug removal are summarized in Table 2.

## 6. Patient-Related Determinants in Critical Illness

Drug exposure during renal replacement therapy is influenced not only by the extracorporeal prescription but also by the rapidly changing physiology of critical illness. Sepsis, shock, fluid resuscitation, capillary leak, hypoalbuminemia, organ dysfunction, and changes in residual kidney function may alter drug distribution and clearance over short periods. Consequently, a dose that is appropriate at CRRT initiation may become inadequate as the patient’s clinical condition evolves [3,4,6,7].

Residual kidney function remains an important component of total drug clearance during RRT and should not be assumed to be negligible. Renal recovery may occur while CRRT is still ongoing, whereas progressive loss of residual function may increase drug exposure [4,7]. Vancomycin is a clinically relevant example, as residual diuresis has been shown to contribute to interindividual variability in clearance during CRRT [11].

Fluid resuscitation, systemic inflammation, and capillary leak may increase the apparent volume of distribution of hydrophilic drugs, particularly during the early phase of sepsis and shock. Hypoalbuminemia may further modify the unbound fraction of highly protein-bound drugs, affecting distribution, extracorporeal removal, and interpretation of total plasma concentrations [6,10]. Hepatic dysfunction may simultaneously reduce non-renal clearance and increase the risk of accumulation for drugs that depend substantially on metabolism or biliary elimination [4,6,10].

These patient-related determinants should be interpreted together with the actual delivered CRRT prescription and TDM when available. Figure 1 summarizes the interaction between drug-related, CRRT-related, and patient-related determinants of drug exposure.

Drug exposure results from the interaction between drug-specific, treatment-related, and patient-related factors, with different implications for loading-dose selection, maintenance dosing, and repeated reassessment.

CRRT, continuous renal replacement therapy; IHD, intermittent hemodialysis; PK/PD, pharmacokinetic/pharmacodynamic; RRT, renal replacement therapy; SLED, sustained low-efficiency dialysis; TDM, therapeutic drug monitoring.

## 7. Drug Classes and Dosing Implications

Drug dosing during CRRT cannot be approached uniformly across pharmacological classes. The clinical relevance of extracorporeal removal depends on the physicochemical and pharmacokinetic characteristics of each drug, the contribution of residual renal and non-renal clearance, the delivered CRRT prescription, and the therapeutic target. These factors are particularly important for drugs with narrow therapeutic windows, concentration- or time-dependent efficacy, active renally eliminated metabolites, or substantial pharmacokinetic variability. The following sections focus on selected drug classes commonly encountered in critically ill adults and for which CRRT may have clinically relevant dosing implications [3,4,7,10].

The strength of evidence differs between drug classes and individual agents. Therefore, statements based on limited CRRT-specific data, indirect evidence, expert opinion, or pharmacokinetic principles are presented as practical considerations rather than definitive clinical recommendations.

### 7.1. Antimicrobial Therapy

Antimicrobial dosing is particularly challenging during RRT because critical illness and extracorporeal clearance may alter both drug distribution and elimination. Initial dosing should prioritize early achievement of adequate exposure, whereas maintenance dosing should integrate residual kidney function, delivered CRRT intensity, pathogen susceptibility, clinical response, and therapeutic drug monitoring (TDM) when available [3,5,7].

#### 7.1.1. Beta-Lactams

Beta-lactams are predominantly hydrophilic, and many have low-to-moderate protein binding and a substantial renal contribution to total clearance. These properties make several agents susceptible to clinically relevant removal during CRRT. Their antibacterial activity is time-dependent and is primarily related to the duration for which the unbound drug concentration remains above the minimum inhibitory concentration (fT > MIC) [3,5]. Critical illness may increase the apparent volume of distribution, while residual renal function and higher delivered CRRT intensity may increase elimination, creating a substantial risk of underexposure [3,5,6,7,14].

Loading doses should generally not be empirically reduced solely because RRT has been initiated, particularly in severe infection or septic shock. Maintenance dosing should subsequently be adapted to the actual delivered CRRT prescription and changes in residual kidney function [3,5,7]. Extended or continuous infusion can increase the probability of maintaining concentrations above the pharmacodynamic target and is particularly useful when high clearance, less susceptible pathogens, or marked pharmacokinetic variability are present [3,14,15]. Beta-lactam TDM can provide additional guidance when available, particularly in patients with unstable renal function, high CRRT intensity, treatment interruptions, difficult-to-treat infections, or an uncertain pharmacodynamic target. Model-informed precision dosing and Bayesian methods are increasingly being investigated for beta-lactams, including piperacillin and meropenem. These approaches may improve individualized prediction of exposure, but evidence for consistent improvement in major patient-centered outcomes remains limited; they should therefore be presented as dosing-support tools rather than established outcome-improving interventions [16,17,18]. Meropenem illustrates the interaction between these factors particularly well. It has low protein binding, a relatively small volume of distribution, and substantial renal elimination, making extracorporeal clearance clinically relevant [5,14]. Maintenance requirements during CRRT vary with effluent intensity, residual kidney function, organism susceptibility, and the PK/PD target. Fixed empirical dose reduction may therefore result in inadequate exposure, whereas prolonged infusion and individualized dosing provide a more rational approach when the risk of underdosing is high [14,18].

#### 7.1.2. Vancomycin

Vancomycin exposure during CRRT is highly variable. Extracorporeal clearance depends on treatment intensity, membrane characteristics, effective treatment time, and circuit interruptions, while even limited residual kidney function may contribute significantly to total clearance [3,7,11]. Residual diuresis has been identified as an important contributor to interindividual variability in vancomycin pharmacokinetics during CRRT [11]. Loading-dose selection should focus on achieving adequate early exposure rather than on renal dysfunction alone. Subsequent maintenance dosing should be individualized according to measured exposure and changes in residual renal and extracorporeal clearance [3,7,11]. For serious methicillin-resistant Staphylococcus aureus infections, current consensus recommendations favor AUC-guided monitoring, with an AUC24/MIC target of 400–600 when the MIC is assumed to be 1 mg/L. Trough concentration alone is no longer considered the preferred monitoring strategy for these infections [19]. Because CRRT settings and residual kidney function can change rapidly, a vancomycin regimen that initially achieves the target may become inadequate within a short period. Repeated assessment is therefore more appropriate than assuming that a fixed CRRT-specific dose will remain valid throughout treatment [7,11].

A recent systematic review of 12 population pharmacokinetic studies of vancomycin during CRRT highlighted substantial variability in pharmacokinetic estimates and the influence of effluent intensity, residual diuresis, and patient-specific factors. Limited external model validation further emphasizes the need for cautious interpretation of model-derived dosing estimates and individualized dosing guided by therapeutic drug monitoring [20].

#### 7.1.3. Aminoglycosides

Aminoglycosides have low protein binding, relatively small volumes of distribution, and predominantly renal elimination, making them readily accessible to extracorporeal removal. Their antibacterial activity is concentration-dependent, whereas toxicity is related to excessive and prolonged exposure [5,6]. This creates two competing dosing concerns during RRT: an inadequate initial dose may fail to achieve an appropriate peak concentration, while inappropriate maintenance dosing or insufficient extension of the dosing interval may increase toxicity [5,6]. The initial dose should therefore aim to achieve the required pharmacodynamic exposure rather than being automatically reduced because of renal failure. Subsequent administration should be individualized according to measured drug concentrations, residual kidney function, delivered CRRT intensity, and treatment interruptions. Peak and trough concentrations, or pharmacokinetic modeling when available, are particularly useful because conventional dose-reduction tables cannot adequately account for the rapidly changing clearance seen during critical illness and CRRT [5,6,21].

#### 7.1.4. Linezolid

Linezolid differs from many other antimicrobials used during CRRT because its extracorporeal removal is variable and non-renal clearance remains important. Standard dosing may result in insufficient exposure in some critically ill patients, whereas excessive exposure, particularly during prolonged therapy and impaired elimination, may increase the risk of hematologic toxicity, including thrombocytopenia [22]. RRT modality alone is therefore not sufficient to determine whether the dose should be reduced or increased. TDM may be useful when available, particularly during prolonged treatment, marked pharmacokinetic variability, suspected toxicity, or uncertain clinical response. Expert consensus supports TDM for linezolid and highlights the wide variability in exposure under conventional dosing [22,23]. TDM may improve dose individualization and pharmacokinetic target attainment, but this should not be interpreted as definitive evidence of improved patient-centered outcomes, such as mortality, treatment failure, toxicity, duration of organ support, or length of stay [15,16,19,23].

#### 7.1.5. Fluoroquinolones

The effect of RRT on fluoroquinolones varies considerably between individual agents because renal elimination, protein binding, and volume of distribution differ within the class. Ciprofloxacin and levofloxacin may undergo clinically relevant extracorporeal removal, particularly at higher CRRT intensities. Their antibacterial activity is exposure-dependent, and inadequate maintenance dosing may compromise pharmacodynamic target attainment. Dosing should therefore consider organism susceptibility, residual kidney function, RRT modality, and delivered treatment intensity rather than relying solely on conventional renal dose-reduction tables [3,5,6,7]. Fluoroquinolone TDM is considerably less available than monitoring for vancomycin or aminoglycosides, limiting routine concentration-guided adjustment. Model-informed dosing has been investigated for ciprofloxacin, although evidence for improvement in major clinical outcomes remains uncertain [16].

### 7.2. Antifungal Agents

Antifungal drugs have markedly different pharmacokinetic profiles, and the effect of RRT should not be generalized across the class. Fluconazole has low protein binding, a relatively small volume of distribution, and substantial renal elimination. Consequently, it may be efficiently removed during CRRT, and inadequate maintenance dosing can result in underexposure [3,24]. Population pharmacokinetic data in critically ill patients demonstrate a strong influence of renal function and CRRT on fluconazole clearance and substantial variability in target attainment [24]. The loading dose of fluconazole should primarily reflect the required exposure and volume of distribution rather than renal function. Maintenance dosing must subsequently account for CRRT intensity, residual kidney function, organism susceptibility, and the pharmacodynamic target. Available pharmacokinetic studies suggest that conventional dose reductions used for renal failure without RRT may be inadequate in patients receiving CRRT [3,24]. Echinocandins, including anidulafungin, caspofungin, and micafungin, are highly protein-bound and undergo minimal renal elimination. Direct extracorporeal removal is therefore generally limited, and routine dose reduction solely because of CRRT is usually unnecessary [25]. However, critical illness itself may produce substantial pharmacokinetic variability, and standard dosing does not guarantee target attainment in every patient. This distinction is important: limited dialyzability does not necessarily imply predictable exposure [25]. For the parent drug, clinically relevant extracorporeal removal of voriconazole appears limited on pharmacokinetic grounds, and dosing is influenced more strongly by nonlinear pharmacokinetics, hepatic metabolism, drug interactions, and interindividual variability than by extracorporeal clearance. TDM is therefore particularly relevant [26]. The intravenous formulation contains sulfobutylether-β-cyclodextrin (SBECD), an excipient that may accumulate in kidney failure and can be removed to some extent by CRRT; this carrier should be considered separately from voriconazole parent-drug clearance [3,27].

### 7.3. Antiviral Agents

Evidence for antiviral dosing during CRRT is less extensive than for antibacterial therapy and remains highly drug-specific. Acyclovir and ganciclovir are predominantly renally eliminated, have relatively low protein binding, and can undergo clinically important extracorporeal clearance [28]. Dose reduction is necessary when intrinsic renal clearance falls, but excessive reduction during CRRT may also result in inadequate exposure. CRRT modality, effluent intensity, treatment interruptions, and residual kidney function should therefore be considered when selecting maintenance regimens [5,28]. The evidence base for modern antivirals during CRRT remains more limited. Remdesivir and its metabolites have different elimination pathways. Current recommendations allow standard remdesivir dosing across stages of renal impairment, including patients receiving dialysis, although CRRT-specific pharmacokinetic evidence remains limited [28]. Antiviral dosing should therefore remain drug-specific, and statements based on limited pharmacokinetic data should not be generalized to the entire class [28].

### 7.4. Analgesics and Sedatives

Analgesic and sedative dosing during RRT is influenced by kidney dysfunction, extracorporeal clearance, and the pharmacokinetic characteristics of the parent drug and its metabolites. For several analgesic and sedative agents, direct CRRT-specific pharmacokinetic evidence is limited; therefore, some statements in this section are based on known pharmacokinetic properties, metabolite behavior, and cautious interpretation of available RRT data. However, active or potentially toxic renally eliminated metabolites may accumulate during AKI and may be affected differently by intermittent and continuous RRT modalities [10,29].

#### 7.4.1. Opioids

Morphine undergoes hepatic glucuronidation to morphine-3-glucuronide and morphine-6-glucuronide, both of which depend substantially on renal elimination. Accumulation of these metabolites during kidney dysfunction may contribute to prolonged sedation, respiratory depression, and neurotoxicity [10,29]. The parent compound has a relatively large volume of distribution and is less accessible to extracorporeal removal, whereas the more water-soluble glucuronide metabolites may be removed to a greater extent. Because contemporary data on morphine disposition during CRRT remain limited, careful dose titration and clinical monitoring are preferable to fixed dose-reduction schemes [29]. Fentanyl is highly lipophilic, extensively protein-bound, and characterized by a large volume of distribution, making clinically relevant extracorporeal removal unlikely. Its metabolites are not considered clinically active, and extracorporeal clearance represents only a minor component of total fentanyl elimination [10,29]. Based on its pharmacokinetic profile and the limited available RRT-specific evidence, CRRT alone does not provide a strong rationale for routine empirical dose reduction. Nevertheless, prolonged administration, hepatic dysfunction, and changes in critical illness may alter exposure, and dosing should remain guided by analgesic requirements, sedation depth, and adverse effects [10,29]. Hydromorphone is predominantly metabolized in the liver to hydromorphone-3-glucuronide, which is eliminated through the kidneys and may accumulate during renal dysfunction. Evidence regarding its clearance during CRRT remains limited, and intermittent hemodialysis data cannot be directly extrapolated to continuous modalities. Dose titration should therefore be based on clinical response, with monitoring for both inadequate analgesia and metabolite-related toxicity [10,29].

#### 7.4.2. Non-Opioid Analgesics

Acetaminophen is predominantly metabolized hepatically, with only a small proportion excreted unchanged by the kidneys. Although the parent drug is dialyzable, routine dose adjustment is generally not required solely on the basis of AKI. Extracorporeal removal becomes clinically relevant mainly in selected severe intoxications; evidence from overdose should therefore not be extrapolated directly to routine analgesic dosing during CRRT [29]. Ketamine is highly lipophilic, has a large volume of distribution, and undergoes extensive hepatic metabolism. Extracorporeal removal of the parent drug is therefore limited, and dosing should be guided primarily by clinical response, hemodynamic effects, hepatic function, and the consequences of prolonged administration [29].

#### 7.4.3. Sedatives

Midazolam is predominantly metabolized in the liver, but renal dysfunction substantially affects the disposition of its metabolites. The parent compound is poorly removed by RRT, whereas its more water-soluble hydroxylated and glucuronidated metabolites are more susceptible to extracorporeal removal. In particular, 1-hydroxymidazolam-glucuronide may accumulate during kidney dysfunction while also being cleared during CRRT, contributing to substantial variability in sedative exposure. Midazolam should therefore be titrated to the observed depth of sedation and reassessed as kidney function and RRT intensity change [10,29,30].

Based on its high lipophilicity, extensive protein binding, and large volume of distribution, clinically relevant extracorporeal removal of propofol is unlikely, although direct CRRT-specific evidence remains limited. Dosing should therefore be titrated to the desired sedation level and patient tolerance, with attention to cumulative exposure and complications of prolonged or high-dose administration [29,31].

Dexmedetomidine undergoes predominantly hepatic metabolism, is extensively protein-bound, and has minimal renal elimination of unchanged drug. On pharmacokinetic grounds, direct effects of kidney dysfunction and extracorporeal clearance on dexmedetomidine exposure are expected to be limited; however, direct CRRT-specific data are sparse, and dosing should remain guided by sedation target and clinical tolerance, particularly bradycardia and hypotension [29,31].

### 7.5. Anticoagulants and Antithrombotic Therapy

Systemic anticoagulant therapy should be distinguished from anticoagulation used specifically to maintain extracorporeal circuit patency. The latter, including regional citrate anticoagulation, is determined primarily by the CRRT prescription and circuit strategy and falls outside the scope of drug-dose adjustment discussed here. Low-molecular-weight heparins are partly dependent on renal elimination, raising concern for altered exposure in severe kidney dysfunction. However, the effect of CRRT on low-molecular-weight heparin activity cannot be predicted solely from renal function or molecular size [32,33]. In a prospective study of prophylactic enoxaparin in critically ill patients, CVVHDF produced only a modest effect on anti-Xa activity, and the findings did not support routine dose escalation simply because CVVHDF was being used [32]. Anti-Xa activity can be used to characterize LMWH exposure in selected clinical settings, but routine anti-Xa-guided dose adjustment is not universally recommended in patients with severe renal dysfunction. In particular, the ASH guideline suggests against routine anti-Xa monitoring to guide LMWH dose adjustment in patients with creatinine clearance <30 mL/min treated for VTE [33]. Dose decisions should integrate the indication for anticoagulation, bleeding and thrombotic risks, renal function, body weight, and the specific anticoagulant rather than assuming a uniform CRRT effect. Evidence supporting RRT-specific dose changes for many systemic anticoagulants remains limited, and recommendations should therefore remain agent-specific [32,33].

### 7.6. Psychotropic Drugs

CRRT-specific evidence for most antidepressants and other chronic psychotropic medications is limited [10]. Tricyclic antidepressants have large volumes of distribution and extensive protein binding, making meaningful extracorporeal removal unlikely. Similarly, many selective serotonin reuptake inhibitors undergo predominantly hepatic metabolism and have pharmacokinetic characteristics that limit dialysis clearance. In the absence of drug-specific CRRT evidence, routine dose modification solely because of CRRT is difficult to justify for most antidepressants; clinical toxicity, hepatic function, drug interactions, and adverse-effect profiles should guide interpretation [10,34]. Benzodiazepines used for ICU sedation are discussed separately because the accumulation and extracorporeal removal of their active metabolites may be clinically relevant [10,30].

### 7.7. Antidiabetic Agents

Oral glucose-lowering therapy in critically ill patients requires a different approach from stable outpatient management. In patients with severe AKI or receiving CRRT, the decision to continue or discontinue an antidiabetic agent is frequently determined more by acute illness, hemodynamic instability, nutritional status, organ dysfunction, and adverse-effect risk than by extracorporeal drug clearance alone [35]. Metformin is eliminated predominantly through the kidneys and may accumulate in severe renal dysfunction, particularly in the presence of shock, tissue hypoperfusion, or other factors predisposing to lactic acidosis. It is generally withheld in critically ill patients with significant AKI or hemodynamic instability [35]. In severe metformin-associated lactic acidosis, extracorporeal therapy may be used to enhance elimination and correct acid–base abnormalities, but this toxicological indication should be distinguished from routine therapeutic dosing during CRRT [36]. Sulfonylureas are highly protein-bound and therefore are not readily removed by standard hemodialysis, and CRRT-specific data remain limited [37]. Their main clinical concern in kidney dysfunction is prolonged hypoglycemia resulting from reduced drug or metabolite elimination rather than excessive extracorporeal removal. In the critically ill patients, frequent glucose monitoring and avoidance of prolonged hypoglycemia are more relevant than attempting to compensate for presumed CRRT clearance [35,37]. Sodium-glucose cotransporter 2 (SGLT2) inhibitors require particular caution in critically ill patients. The principal issue during severe acute illness is generally whether therapy should be continued rather than how CRRT modifies drug clearance. KDIGO 2024 considers it reasonable to withhold SGLT2 inhibitors during prolonged fasting, surgery, or critical medical illness, and also notes that continuation below an eGFR of 20 mL/min/1.73 m^2^ is reasonable unless kidney replacement therapy is initiated [38]. Current ADA inpatient guidance similarly recommends avoiding SGLT2 inhibitors in severe illness, ketonemia or ketonuria, prolonged fasting, and surgical settings [35].

### 7.8. General Dosing Implications Across Drug Classes

Across drug classes, the clinical impact of RRT depends on the relative contribution of extracorporeal clearance to total drug elimination. Drugs with low protein binding, low-to-moderate volume of distribution, hydrophilicity, and substantial renal elimination are generally more susceptible to CRRT-related removal, whereas highly lipophilic or extensively protein-bound drugs with large volumes of distribution are less directly affected [3,4,6,10,29]. In Table 3A,B, CRRT-specific considerations are separated from IHD/SLED considerations where possible. Because the level of evidence differs between drugs and renal replacement therapy modalities, some statements are supported by CRRT-specific pharmacokinetic data or TDM-based recommendations, whereas others rely on broader RRT evidence, limited observational data, in vitro findings, expert consensus, or pharmacokinetic extrapolation. Where direct modality-specific evidence is limited, the statements should be interpreted as practical considerations rather than fixed dosing recommendations.

The qualitative descriptors used for extracorporeal removal (“high,” “moderate–high,” “variable,” “variable–high,” “low,” “very low,” or “negligible”) are intended as approximate clinical categories rather than quantitative clearance thresholds. “High” indicates that extracorporeal removal is expected to be clinically relevant in typical extracorporeal treatment settings; “moderate–high” or “variable–high” indicates potentially relevant removal that depends strongly on treatment intensity, residual kidney function, membrane characteristics, and patient factors; “variable” indicates inconsistent or context-dependent removal; and “low,” “very low,” or “negligible” indicates that extracorporeal treatment is unlikely to substantially affect parent-drug exposure, although metabolites or critical illness-related changes may still be clinically relevant.

Because Table 3A,B are intended to support clinical reasoning rather than provide fixed dosing prescriptions, published source-referenced CRRT regimens for selected drugs are summarized separately in Supplementary Table S2. These regimens should be interpreted as reported starting points from specific populations and CRRT settings, not as universally applicable recommendations.

This review does not aim to provide an exhaustive catalogue of all ICU drugs used during CRRT. Drugs not discussed in detail, including antiepileptics, vasopressors, inotropes, insulin, proton pump inhibitors, polymyxins including colistin, tigecycline, daptomycin, cefiderocol, newer beta-lactam/beta-lactamase inhibitor combinations, and amphotericin formulations, should be approached on an agent-specific basis. Dosing decisions should consider the dominant elimination pathway, protein binding, volume of distribution, therapeutic index, residual kidney function, delivered CRRT intensity, toxicity risk, and TDM availability when relevant [3,4,5,6,7,10].

## 8. Therapeutic Drug Monitoring and Individualized Dosing

Therapeutic drug monitoring (TDM) is an important component of individualized dosing during renal replacement therapy because pharmacokinetic variability in critically ill patients cannot be reliably captured by renal function estimates or RRT prescription alone. Because these variables may change simultaneously during critical illness, measured concentrations should be interpreted in clinical context rather than in isolation. TDM is therefore most useful when the relationship between drug exposure and efficacy or toxicity is reasonably well defined and when measured concentrations can lead to a clinically meaningful dose adjustment [3,4,6,7]. The role of TDM differs between drug classes. For vancomycin, aminoglycosides, and selected other antimicrobials, concentration-guided dosing is already well established. For serious MRSA infections, vancomycin monitoring should preferentially be based on AUC rather than trough concentration alone, with repeated reassessment when residual kidney function or CRRT intensity changes [11,19]. Aminoglycoside dosing similarly requires concentration-based assessment because an adequate initial peak must be balanced against the risk of excessive residual exposure and toxicity [6,21,39]. For linezolid, TDM is not universally available but may be particularly useful when prolonged treatment, altered clearance, thrombocytopenia, or marked pharmacokinetic variability raises concern for either underexposure or toxicity [22,23].

Beta-lactam TDM has received increasing attention in critically ill patients because standard dosing frequently produces highly variable concentrations. Critical illness, renal recovery, augmented or extracorporeal clearance, changes in fluid balance, and interruptions in CRRT may all affect target attainment. Measurement of beta-lactam concentrations can therefore be useful when the risk of underexposure is high, particularly in severe infection, less susceptible pathogens, high or changing RRT intensity, or when prolonged or continuous infusion is used [7,15]. However, optimal PK/PD targets and the level of exposure associated with toxicity are not identical across beta-lactams, and routine availability of rapid assays remains limited. TDM should therefore support, rather than replace, clinical and microbiological assessment [15].

A further limitation of conventional TDM is that a measured concentration reflects exposure at a particular point in time and may be difficult to translate directly into a new dosing regimen when pharmacokinetics are changing rapidly. Model-informed precision dosing (MIPD) attempts to address this limitation by combining population pharmacokinetic models, patient-specific covariates, measured drug concentrations, and Bayesian forecasting to estimate individual pharmacokinetic parameters and predict subsequent exposure. In principle, this approach is particularly attractive in CRRT, where clearance depends on multiple time-varying patient and treatment variables [7,17].

The usefulness of MIPD depends on the quality and external validity of the underlying pharmacokinetic model. Available dosing platforms differ in the drugs and models supported, their ability to incorporate measured concentrations, and their integration into clinical workflow [17,40,41]. External evaluations have demonstrated substantial differences in predictive performance between published pharmacokinetic models, emphasizing the need for validation in the population and RRT setting in which they are applied [17]. Although MIPD may improve pharmacokinetic prediction and target attainment, evidence for consistent improvement in major patient-centered outcomes remains limited. External evaluations of CRRT pharmacometric models have also shown substantial variability in model performance, supporting cautious implementation, clinical oversight, and local validation [16,17].

TDM and MIPD should therefore be integrated into repeated clinical reassessment rather than used as isolated dosing tools. Measured concentrations, clinical and microbiological response, residual organ function, and the actual delivered CRRT should be considered together when adjusting subsequent doses [3,4,6,7,16,17].

Table 4 summarizes practical monitoring considerations by drug class. These considerations should be interpreted in clinical context and adapted to drug-specific evidence, assay availability, local protocols, and patient-specific factors rather than as prescriptive recommendations.

## 9. Practical Approach to Drug Dosing During CRRT

Drug dosing during CRRT should be approached as a dynamic process rather than as a fixed adjustment based on renal function alone. The initial prescription should integrate the patient’s residual kidney function, the RRT modality and actual delivered treatment, drug-specific pharmacokinetic and pharmacodynamic characteristics, and the therapeutic target. Because each of these components may change during critical illness, dosing decisions require repeated reassessment throughout treatment [4,7,13,42].

This framework does not propose new pharmacokinetic principles and was not developed through a Delphi process, expert panel, retrospective validation, or prospective testing. Its purpose is to organize established dosing determinants into a CRRT-focused bedside sequence. Compared with earlier stepwise approaches, the framework places greater emphasis on delivered rather than prescribed CRRT intensity, separation of loading-dose and maintenance-dose decisions, interpretation of evidence according to modality and drug-specific data, and repeated reassessment as patient status and circuit performance change.

The following author-proposed stepwise approach is intended as a conceptual framework for clinical reasoning in critically ill adults receiving CRRT. It has not been formally validated or prospectively evaluated and should not be interpreted as a prescriptive clinical algorithm or clinical guideline.

Step 1. Assess residual kidney function.

Residual renal clearance should be considered even when RRT is ongoing. Urine output, trends in renal function, and evidence of renal recovery or deterioration may provide clinically relevant information, particularly for drugs predominantly eliminated by the kidneys [4,7]. Residual kidney function should not be assumed to be negligible simply because CRRT has been initiated, as even limited native clearance may contribute substantially to total drug elimination for selected agents [7,11].

Step 2. Identify the CRRT modality and treatment schedule.

Within a CRRT-focused framework, the relevant technical distinction is the CRRT mode, such as CVVH, CVVHD, or CVVHDF, together with delivered treatment time, interruptions, and changes in circuit function. Evidence from intermittent or prolonged intermittent modalities should be interpreted as contextual rather than directly interchangeable with CRRT [3,7,13,42].

Step 3. Document the actual technical prescription.

Relevant parameters include blood flow, dialysate flow, total ultrafiltration flow, replacement-fluid rate and location, effluent flow, membrane characteristics, treatment duration, and circuit interruptions. Prescribed CRRT intensity should be distinguished from the treatment actually delivered because downtime, procedures, alarms, filter clotting, and circuit replacement may substantially reduce effective extracorporeal clearance [8,12,13,43].

Step 4. Evaluate the drug’s pharmacokinetic and pharmacodynamic characteristics.

Assess whether the drug profile supports clinically relevant extracorporeal removal, considering protein binding, molecular size, volume of distribution, hydrophilicity, renal elimination, and the relevant PK/PD target. These variables should be interpreted together rather than as isolated predictors [3,4,6,7].

Step 5. Determine the loading dose independently from maintenance clearance whenever appropriate.

Loading-dose decisions should mainly reflect target exposure and volume of distribution. In severe infection or sepsis, the initial dose should not be empirically reduced solely because kidney function is impaired or CRRT has been initiated [3,5,6,7].

Step 6. Adjust the maintenance regimen according to total drug clearance.

Maintenance dosing should be adjusted according to total clearance, including residual renal, extracorporeal, and non-renal clearance. Changes in CRRT intensity, renal recovery, hepatic dysfunction, circuit performance, or interruptions may require adjustment of dose, interval, infusion duration, or a combination of these approaches [3,4,7]. For time-dependent antimicrobials, prolonged or continuous infusion may be preferable when the risk of underexposure is high [14,15].

Step 7. Use therapeutic drug monitoring when clinically appropriate and available.

TDM is particularly valuable when exposure is difficult to predict, the therapeutic window is narrow, or validated concentration targets are available. Vancomycin, aminoglycosides, selected beta-lactams, and linezolid are important examples [15,19,21,23,39]. Measured concentrations should be interpreted in relation to the time of sampling, RRT status at the time of measurement, recent treatment interruptions, and changes in residual kidney function [7,11]. Bayesian or model-informed dosing may provide additional support where validated tools are available, but these approaches should complement rather than replace clinical assessment [16,40,41].

Step 8. Reassess the regimen whenever the patient or RRT prescription changes.

Drug dosing should be reconsidered after clinically meaningful changes in urine output, renal recovery, fluid balance, albumin concentration, hepatic function, hemodynamic status, CRRT intensity, membrane or filter replacement, prolonged downtime, or therapeutic response. A regimen selected at RRT initiation should not be assumed to remain appropriate throughout the entire course of critical illness [4,6,7,12,13].

This framework emphasizes repeated reassessment of residual renal, extracorporeal, and non-renal clearance rather than fixed dose adjustment based on AKI or prescribed CRRT intensity alone [3,4,6,7,12,13]. A practical stepwise approach is illustrated in Figure 2.

### Worked Examples of Applying the CRRT Dosing Framework

Example 1: Hydrophilic beta-lactam during high-intensity CRRT. In a critically ill patient receiving a beta-lactam during high-intensity CRRT, the framework separates the loading dose from the maintenance regimen. The loading dose should generally be preserved to support early PK/PD target attainment, while maintenance dosing is reassessed according to delivered effluent dose, residual urine output, pathogen susceptibility, and the feasibility of prolonged or continuous infusion. When delivered CRRT clearance is high or residual kidney function persists, the framework supports avoiding automatic dose reduction and reassessing exposure with measured concentrations when available, or with clinical and microbiological response when TDM is unavailable [3,5,7,15].

Example 2: Vancomycin during interrupted CRRT. In a patient receiving vancomycin during CRRT, a concentration below or above target should prompt reassessment of total clearance rather than adjustment based on AKI alone. The framework directs review of delivered effluent dose, residual urine output, circuit downtime, and sampling time. If exposure is low despite severe AKI, maintenance dosing may need to be maintained or increased when CRRT delivery is high or residual clearance persists. If exposure is high after CRRT interruption or loss of residual clearance, the next dose may need to be delayed or reduced, with repeat TDM [7,11,19].

The approach integrates residual kidney function, CRRT modality and delivered treatment, drug-specific PK/PD characteristics, loading- and maintenance-dose selection, therapeutic drug monitoring, and repeated reassessment as patient status and CRRT delivery evolve. This framework has not been prospectively validated and should not be interpreted as a prescriptive clinical algorithm or clinical guideline.

CRRT, continuous renal replacement therapy; IHD, intermittent hemodialysis; PK/PD, pharmacokinetic/pharmacodynamic; RRT, renal replacement therapy; SLED, sustained low-efficiency dialysis; TDM, therapeutic drug monitoring.

## 10. Bioengineering and Digital Support for Individualized Dosing During CRRT

Continuous renal replacement therapy generates treatment-related data that are directly relevant to extracorporeal drug clearance, including delivered effluent dose, treatment interruptions, and filter changes [12,13,43]. When combined with patient-specific variables and TDM results, these data may support more individualized estimation of drug exposure during critical illness [4,7,12,16].

Digital approaches, including Bayesian forecasting, model-informed precision dosing, machine-learning tools, and digital-twin concepts, may help integrate these dynamic variables into dosing support during CRRT [16,40,41,44,45,46]. However, these approaches remain insufficiently validated for routine clinical use and require external validation, workflow integration, clinician oversight, and prospective evidence of clinically meaningful benefit [16,40,41,47,48].

## 11. Limitations

Several limitations should be acknowledged. This structured narrative review was not designed as a systematic review, and the evidence base remains heterogeneous across RRT modalities, treatment intensity, membranes, patient populations, pharmacokinetic methods, and therapeutic targets. CRRT-specific data remain limited for several agents; therefore, some practical statements rely on pharmacokinetic principles, indirect evidence, or cautious extrapolation and should not be interpreted as prescriptive clinical guidance. The drugs included in the practical tables are representative rather than exhaustive, and the pharmacokinetic values presented are approximate and source-dependent. This caution is further supported by evidence that antimicrobial dosing recommendations during CRRT may differ substantially between commonly used drug-information databases, reinforcing that tabulated regimens should be interpreted as source-reported examples rather than fixed dosing recommendations [49]. The proposed eight-step framework has not undergone formal expert consensus, retrospective validation, or prospective external validation. Finally, much of the literature focuses on pharmacokinetic exposure or target attainment, while evidence for improvement in major patient-centered outcomes remains limited. 

## 12. Future Perspectives

Future research should focus on reducing the uncertainty that currently limits drug dosing during RRT. More consistent reporting of actual delivered CRRT intensity, treatment interruptions, membrane characteristics, residual kidney function, renal recovery, and pharmacokinetic sampling is needed to improve comparability between studies and the external validity of dosing recommendations [4,12,13,42,43]. Recent CRRT-specific scoping work has further emphasized the need for standardized reporting of treatment-related and patient-related variables, including CRRT modality, delivered effluent dose, downtime, membrane and filter characteristics, residual kidney function, sampling strategy, and relevant patient covariates. The proposed CRRT-PK checklist may help improve comparability between studies and strengthen the evidence base for individualized antimicrobial dosing during CRRT [50]. Prospective pharmacokinetic studies should increasingly combine detailed RRT delivery data with measured drug concentrations, because exposure cannot be inferred reliably from the prescribed extracorporeal treatment alone [7,50,51]. A recent systematic review of population pharmacokinetic studies of vancomycin during CRRT also confirmed marked heterogeneity in reported clearance and volume-of-distribution estimates across models and highlighted effluent intensity, residual diuresis, albumin concentration, and other patient-specific covariates as important determinants of exposure [20]. Importantly, future trials should evaluate clinically meaningful endpoints in addition to pharmacokinetic target attainment, including treatment failure, drug toxicity, antimicrobial resistance, duration of organ support, and patient-centered outcomes [16,20,51].

Future dosing models should also account more explicitly for the dynamic biology of critical illness. Inflammation, capillary leak, hypoalbuminemia, organ dysfunction, and evolving renal recovery may alter pharmacokinetics independently of the extracorporeal circuit. Emerging approaches therefore aim to integrate TDM, biomarkers, clinical covariates, and model-informed dosing rather than treating RRT intensity as the sole determinant of dose adjustment [6,7,51]. Such models may be particularly relevant in sepsis, where both drug disposition and extracorporeal clearance can change substantially over short periods [51].

Drug dosing during transitions between RRT modalities represents another important research gap. Conversion from CRRT to intermittent or prolonged intermittent therapy, temporary interruption of extracorporeal treatment, and recovery of native kidney function may produce abrupt changes in total drug clearance. Future dosing frameworks should therefore address not only stable CRRT prescriptions but also initiation, interruption, modality transition, de-escalation, and discontinuation of RRT [7,13,52].

The integration of TDM, Bayesian forecasting, model-informed precision dosing, and continuously updated RRT data may support more responsive dose adjustment as patient physiology and extracorporeal clearance change [17,40,41,44]. Although most evidence concerns antimicrobials, similar PK/PD approaches are emerging for sedative and anesthetic drugs [53]. Broader implementation will require external validation, workflow integration, clinician oversight, and demonstration of clinical benefit [16,40,41,47,48].

More broadly, this approach is consistent with the concept of personalized physiological medicine in critical care, in which treatment is adapted to the patient’s changing physiological state rather than applied according to fixed schemes [54].

## 13. Conclusions

Drug dosing during CRRT in critically ill patients should not be based solely on eGFR or fixed renal dose-reduction schemes. Instead, it should integrate drug-specific pharmacokinetic properties, residual kidney function, actual delivered CRRT, and non-renal clearance. Loading and maintenance doses should be considered separately, with TDM used when available and dosing reassessed dynamically as patient physiology, kidney function, and CRRT delivery evolve. This individualized approach better reflects the variability in drug exposure during CRRT.

## Figures and Tables

**Figure 1:**
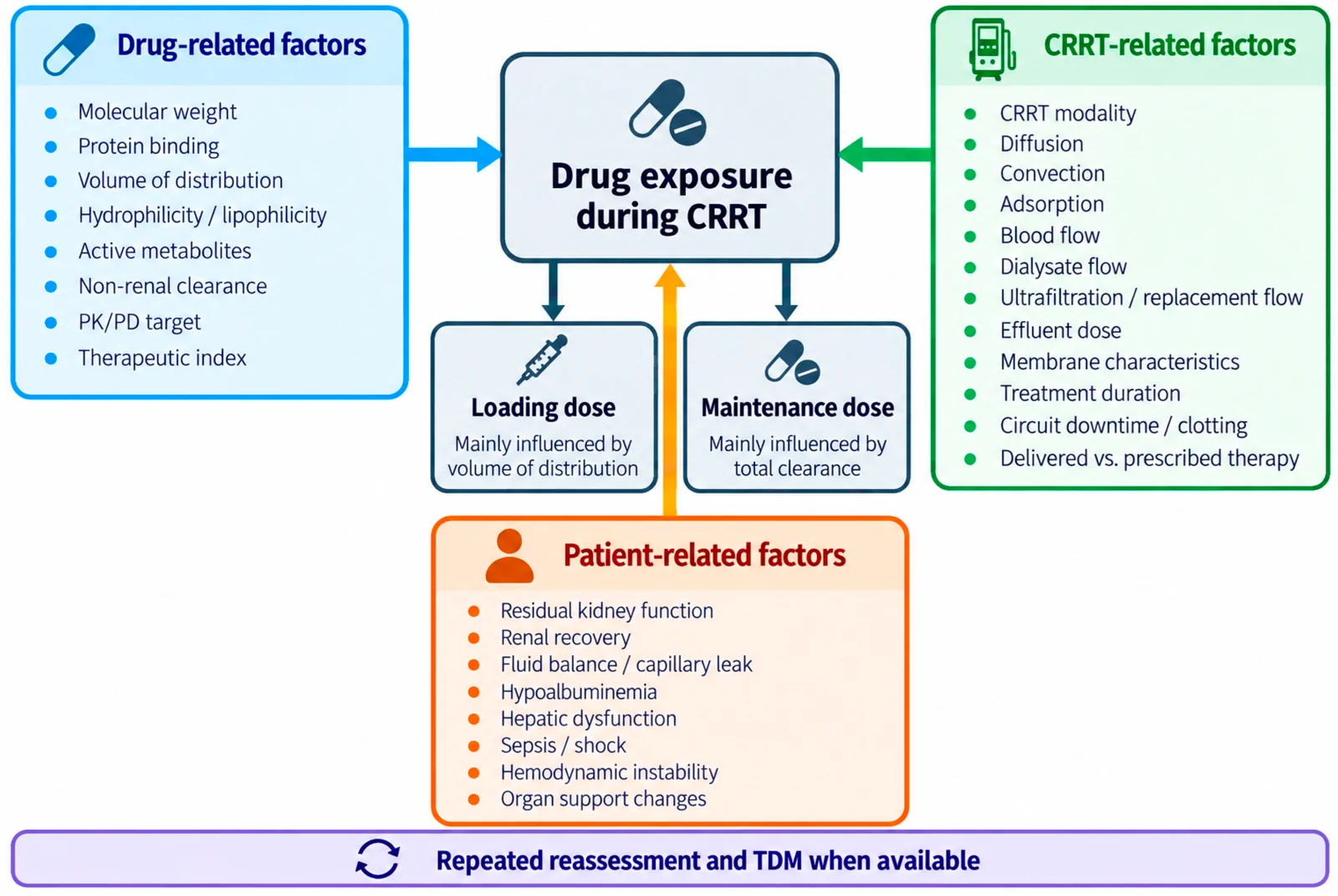
Determinants of drug exposure during continuous renal replacement therapy.

**Figure 2:**
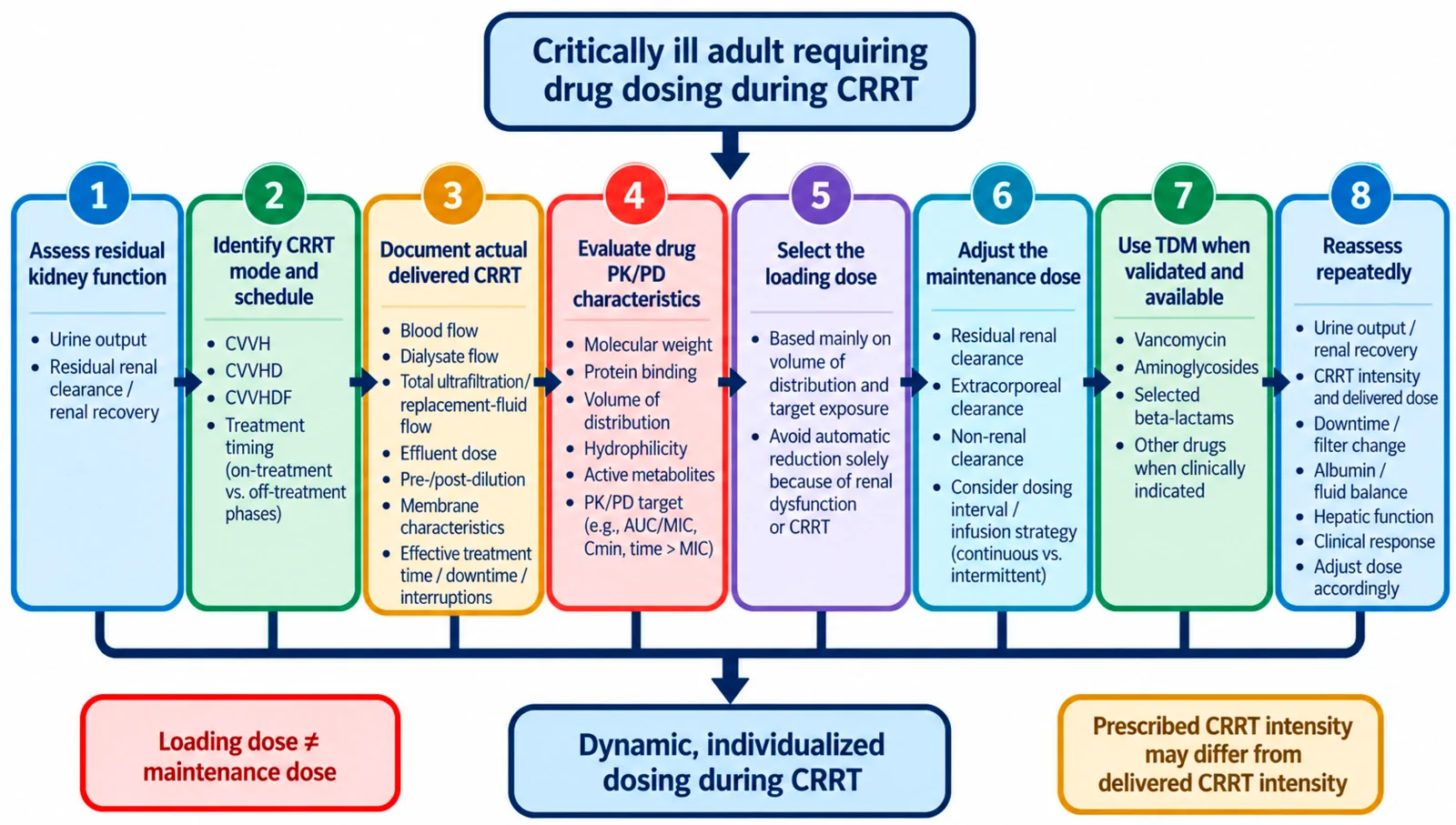
Author-proposed conceptual framework for individualized drug dosing during continuous renal replacement therapy.

**Table 1:** Pharmacokinetic determinants of extracorporeal drug removal.

Property	Effect on Extracorporeal Removal	Practical Implication	Reference(s)
Molecular weight	Lower molecular weight generally favors membrane passage	Interpret together with membrane permeability and protein binding	[3,4,6]
Protein binding	High protein binding reduces removal of the circulating drug	Hypoalbuminemia may increase the unbound fraction	[3,4,6]
Volume of distribution	Low Vd favors extracorporeal removal; high Vd limits it	High-Vd drugs are generally less affected by RRT	[3,4,6]
Hydrophilicity	Generally favors extracorporeal removal	Particularly relevant for hydrophilic antimicrobials	[3,5,6]
Lipophilicity	Tissue distribution limits the circulating drug available for removal	RRT generally has limited effect on highly lipophilic drugs	[3,10]
Residual renal clearance	Adds to extracorporeal clearance	Reassess during renal recovery or changes in urine output	[4,7,11]
Non-renal clearance	Reduces the relative contribution of RRT to total clearance	Hepatic dysfunction may increase accumulation	[4,6,10]
Active/toxic metabolites	Parent drug and metabolites may differ in dialyzability	Consider metabolite accumulation separately	[10]
Therapeutic index/PK/PD target	Does not directly determine removal but determines its clinical relevance	Greater need for TDM when exposure targets are narrow	[3,6,7]

MW, molecular weight; Vd, volume of distribution; RRT, renal replacement therapy; PK/PD, pharmacokinetic/pharmacodynamic; TDM, therapeutic drug monitoring.

**Table 2:** CRRT-related treatment parameters influencing drug clearance.

Parameter	Effect on Extracorporeal Clearance	Practical Implication	Reference(s)
RRT modality	Determines the pattern and mechanism of drug removal	Differentiate between CRRT, IHD, and SLED	[2,8,13]
Blood flow	Influences solute delivery to the filter	Higher blood flow does not necessarily increase drug clearance	[8,12,13]
Dialysate flow	Increases diffusive clearance	Particularly relevant for small, hydrophilic drugs	[3,8]
Ultrafiltration and replacement flow	Increases convective clearance	Consider total convective intensity	[8,12]
Pre- and post-dilution	Predilution reduces effective clearance	Account for replacement-fluid location	[8,12,13]
Effluent flow	Reflects CRRT intensity	Higher delivered effluent flow may increase drug removal	[3,12,13]
Membrane characteristics	Permeability and adsorption may affect drug removal	Consider membrane type when exposure is unexpected	[3,9]
Treatment time and downtime	Determine actual delivered clearance	Interruptions reduce drug removal	[4,12,13]

CRRT, continuous renal replacement therapy; IHD, intermittent hemodialysis; RRT, renal replacement therapy; SLED, sustained low-efficiency dialysis.

**Table 3:** (**A**). Practical dosing considerations for selected antimicrobial drugs during CRRT, with contextual IHD/SLED considerations. (**B**). Practical dosing considerations for selected analgesic and sedative drugs during CRRT, with contextual IHD/SLED considerations.

Drug	MW (Da)	PB (%)	Vd (L/kg)	RRT Removal	CRRT	IHD/SLED	Main Risk	Monitoring	Reference(s)
(**A**)									
Meropenem	383	~2	~0.35	High	Preserve adequate loading dose; adapt maintenance dosing to delivered CRRT; prolonged infusion may improve target attainment	IHD: significant removal; SLED: limited data; consider session duration and intensity	Underexposure	TDM when available; clinical and microbiological response	[3,7,14,15,18]
Piperacillin/tazobactam	518/300	~30/~22	~0.2–0.4	Moderate–high	Adapt maintenance dosing to delivered CRRT intensity; prolonged or continuous infusion may improve exposure	IHD: clinically relevant removal; SLED: limited, session-dependent data	Underexposure	TDM when available	[3,7,15,17]
Vancomycin	1448	Variable	0.47–1.1	Variable–high	Use an adequate loading dose for early exposure; guide maintenance dosing by AUC, CRRT intensity, and residual diuresis	IHD: significant removal with high-flux membranes; SLED: variable and session-dependent removal	Underexposure or toxicity	AUC-guided TDM	[3,7,11,19]
Amikacin	586	<5	0.22–0.50	High	Preserve adequate initial exposure; individualize subsequent doses and dosing intervals	IHD: substantial removal; SLED: limited specific data	Inadequate peak or toxicity	Peak and trough concentrations	[5,39]
Linezolid	338	~30	0.50–0.80	Variable	Routine CRRT-related dose reduction is not automatically required; individualize according to exposure	IHD: variable removal; SLED: limited data	Underexposure or thrombocytopenia	TDM when available; CBC	[22,23]
Fluconazole	306	~12	~0.7	High	Preserve loading dose; maintenance dosing may need to remain relatively high with greater CRRT intensity	IHD: significant removal; SLED: limited, session-dependent evidence	Underexposure	Clinical response; TDM when available	[3,24]
Voriconazole	349	~60	~4	Low for parent drug; SBECD separate	Guided by TDM; parent-drug CRRT removal is limited; consider SBECD separately	IHD: limited parent-drug removal; SLED: limited data	Underexposure or toxicity	TDM	[3,26,27]
(**B**)									
Morphine	285	~20–40	Variable	Parent drug: low; metabolites more relevant	Prefer alternatives when feasible; consider M3G and M6G accumulation; titrate cautiously	IHD: metabolites may be removed; SLED: limited specific data	Sedation, respiratory depression, or neurotoxicity	Analgesia, sedation, and respiratory assessment	[10,29]
Fentanyl	336	~84–87	~3–5	Very low	No routine CRRT-specific dose adjustment; use the lowest effective dose and titrate clinically	IHD: minimal removal; SLED: limited specific data	Accumulation or oversedation	Analgesia and sedation assessment	[10,29]
Hydromorphone	285	~8	~4	Parent drug: limited; metabolite behavior more relevant	Titrate to clinical response; consider H3G accumulation	IHD: variable removal; SLED: limited specific data	Neurotoxicity or oversedation	Clinical assessment	[10,29]
Ketamine	238	Variable	Large	Low	RRT generally has limited direct effect; titrate clinically	Limited dialysis effect; SLED-specific evidence limited	Accumulation or adverse effects	Clinical response	[29]
Midazolam	325	~94–98	~1–3	Parent drug: low; glucuronidated metabolites more removable	Titrate to sedation target; metabolite exposure may change during CRRT	IHD: parent drug poorly removed; SLED: limited specific data	Prolonged or variable sedation	Validated sedation scale	[10,29,30]
Propofol	178	~90–99	Large/variable	Negligible	No CRRT-specific dose adjustment; titrate clinically	Minimal effect of extracorporeal therapy	Cumulative toxicity	Sedation target; clinical monitoring	[29,31]
Dexmedetomidine	200	~93–94	~2	Low	No routine CRRT-specific dose adjustment	Limited extracorporeal effect; SLED-specific evidence limited	Bradycardia or hypotension	Sedation level and hemodynamics	[29,31]

(**A**) AUC, area under the concentration–time curve; CBC, complete blood count; CRRT, continuous renal replacement therapy; IHD, intermittent hemodialysis; MW, molecular weight; PB, protein binding; RRT, renal replacement therapy; SLED, sustained low-efficiency dialysis; TDM, therapeutic drug monitoring; Vd, volume of distribution. **Note:** MW, PB, and Vd values are approximate and may vary between sources and during critical illness. Values were extracted from cited pharmacokinetic studies, drug-specific reviews, and prescribing information when available, and are intended to characterize pharmacokinetic profiles relevant to extracorporeal removal rather than fixed patient-specific values. For combination products, values separated by a slash refer to the individual components. Parent-drug clearance should be distinguished from excipient or carrier removal, as illustrated by voriconazole and its intravenous SBECD carrier. The level of evidence differs between drugs and renal replacement therapy modalities; some statements are supported by CRRT-specific pharmacokinetic data, whereas others rely on broader RRT evidence, TDM-based recommendations, in vitro data, expert consensus, or pharmacokinetic extrapolation. Evidence specific to SLED is limited for several drugs, and IHD/SLED considerations are included only for contextual comparison rather than comprehensive modality-specific dosing guidance. (**B**) CRRT, continuous renal replacement therapy; H3G, hydromorphone-3-glucuronide; IHD, intermittent hemodialysis; M3G, morphine-3-glucuronide; M6G, morphine-6-glucuronide; MW, molecular weight; PB, protein binding; RRT, renal replacement therapy; SLED, sustained low-efficiency dialysis; Vd, volume of distribution. Note: Pharmacokinetic values are approximate and may vary between sources and during critical illness. Values were extracted from cited pharmacokinetic studies, drug-specific reviews, and prescribing information when available. For drugs with wide interindividual variability, qualitative descriptors are used instead of narrow numeric values. Direct CRRT-specific evidence remains limited for several analgesic and sedative drugs; therefore, some statements rely on pharmacokinetic principles, metabolite behavior, limited RRT data, or cautious extrapolation. IHD/SLED considerations are included only for contextual comparison and should not be interpreted as comprehensive modality-specific dosing.

**Table 4:** Practical monitoring considerations by drug class.

Drug or Class	Main Risk During CRRT	Monitoring Consideration	Reference(s)
Beta-lactams	Underexposure or accumulation	TDM when available; clinical and microbiological response	[7,15]
Vancomycin	Underexposure or toxicity	AUC-guided TDM	[19]
Aminoglycosides	Inadequate peak or accumulation	Peak and trough concentrations; PK-guided monitoring	[6,21,39]
Linezolid	Underexposure or thrombocytopenia	TDM when available; blood count	[23]
Fluoroquinolones	Variable exposure	Clinical response; selective TDM	[7,16]
Fluconazole and voriconazole	Underexposure or toxicity	TDM when available, particularly for voriconazole	[24,26]
Echinocandins	Variable exposure	Clinical response	[25]
Opioids	Accumulation or metabolite toxicity	Analgesia, sedation, and respiratory assessment	[10,29]
Sedatives	Prolonged or inadequate sedation	Validated sedation scales; clinical response	[29,31]
LMWH	Bleeding or inadequate anticoagulation	Anti-Xa monitoring when clinically indicated	[32,33]
Antidiabetic agents	Hypoglycemia, acidosis, or ketosis	Glucose and relevant metabolic parameters	[35,38]

AUC, area under the concentration–time curve; LMWH, low-molecular-weight heparin; PK, pharmacokinetic; RRT, renal replacement therapy; TDM, therapeutic drug monitoring.

## Data Availability

No new data were created or analyzed in this study. Data sharing is not applicable to this article.

## Supplementary Materials

The following supporting information can be downloaded at: https://www.mdpi.com/article/10.3390/pharmaceutics18101320/s1; Supplementary Table S1. Literature search framework, representative PubMed search blocks, and supplementary targeted search approaches used for the structured non-systematic narrative review; Supplementary Table S2. Published CRRT dosing regimens reported for selected anti-infective drugs.

## Abbreviations

The following abbreviations are used in this manuscript:

**Abbreviation**	**Definition**
ADA	American Diabetes Association
AKI	Acute kidney injury
ASH	American Society of Hematology
AUC	Area under the concentration–time curve
CBC	Complete blood count
CRRT	Continuous renal replacement therapy
CVVHDF	Continuous venovenous hemodiafiltration
eGFR	Estimated glomerular filtration rate
H3G	Hydromorphone-3-glucuronide
ICU	Intensive care unit
IHD	Intermittent hemodialysis
KDIGO	Kidney Disease: Improving Global Outcomes
LMWH	Low-molecular-weight heparin
M3G	Morphine-3-glucuronide
M6G	Morphine-6-glucuronide
MIC	Minimum inhibitory concentration
MW	Molecular weight
PB	Protein binding
PK/PD	Pharmacokinetics/pharmacodynamics
SBECD	Sulfobutylether-β-cyclodextrin
RRT	Renal replacement therapy
SGLT2	Sodium-glucose cotransporter 2
SLED	Sustained low-efficiency dialysis
TDM	Therapeutic drug monitoring
Vd	Volume of distribution
VTE	Venous thromboembolism
