# Supplementary material for: Drug Dosing During Continuous Renal Replacement Therapy in Critically Ill Patients: A Practical Narrative Review
Source: Pharmaceutics. 2026 Oct 18;18(10):1320. doi: 10.3390/pharmaceutics18101320 (PMC13708363; doi:10.3390/pharmaceutics18101320)
Supplement: Supplementary file 1 [file pharmaceutics-18-01320-s001.zip › pharmaceutics-4598418-supplementary.pdf]

Supplementary Table S1. Literature search framework, representative PubMed search blocks, and supplementary targeted search approaches used for the structured non-systematic narrative review

Source	Search domain	Representative search block	Purpose
PubMed	General RRT/CRRT drug dosing	("renal replacement therapy" OR "continuous renal replacement therapy" OR CRRT OR "intermittent hemodialysis" OR hemodialysis OR haemodialysis OR hemofiltration OR haemofiltration OR hemodiafiltration OR haemodiafiltration OR "sustained low-efficiency dialysis" OR SLED) AND ("drug dosing" OR "dose adjustment" OR "drug clearance" OR pharmacokinetics OR pharmacodynamics OR "therapeutic drug monitoring")	Identification of general literature on drug disposition, pharmacokinetics, pharmacodynamics, therapeutic monitoring, and dosing during RRT
PubMed	Pharmacokinetic determinants of extracorporeal drug removal	("continuous renal replacement therapy" OR CRRT OR "renal replacement therapy") AND ("drug clearance" OR pharmacokinetics OR pharmacodynamics OR "protein binding" OR "volume of distribution" OR hydrophilicity OR lipophilicity OR "molecular weight")	Identification of drug-related determinants of extracorporeal clearance
PubMed	Technical determinants of RRT-related clearance	("continuous renal replacement therapy" OR CRRT OR "renal replacement therapy") AND ("effluent flow" OR "effluent dose" OR "dialysate flow" OR ultrafiltration OR "replacement fluid" OR predilution OR postdilution OR "blood flow" OR membrane OR filter OR adsorption) AND ("drug clearance" OR pharmacokinetics OR dosing)	Identification of RRT-related technical factors affecting drug clearance
PubMed	Antimicrobial dosing	("continuous renal replacement therapy" OR CRRT OR "renal replacement therapy") AND (antimicrobial OR antibiotic OR "beta-lactam" OR carbapenem OR meropenem OR piperacillin OR vancomycin OR aminoglycoside OR linezolid OR fluoroquinolone) AND (dosing OR pharmacokinetics OR pharmacodynamics OR "therapeutic drug monitoring")	Identification of antimicrobial dosing and pharmacokinetic studies
PubMed	Antifungal and antiviral therapy	("continuous renal replacement therapy" OR CRRT OR "renal replacement therapy") AND (antifungal OR fluconazole OR voriconazole OR echinocandin OR acyclovir OR ganciclovir OR antiviral OR remdesivir) AND (dosing OR pharmacokinetics OR clearance)	Identification of evidence for selected antifungal and antiviral agents
PubMed	Analgesics, sedatives, and other selected drug classes	("continuous renal replacement therapy" OR CRRT OR "renal replacement therapy" OR hemodialysis OR haemodialysis) AND (analgesic OR opioid OR morphine OR fentanyl OR hydromorphone OR ketamine OR midazolam OR propofol OR dexmedetomidine OR anticoagulant OR antidiabetic) AND (dosing OR pharmacokinetics OR clearance)	Identification of dosing considerations for non-antimicrobial drug classes
PubMed	Therapeutic drug monitoring and model-informed dosing	("continuous renal replacement therapy" OR CRRT OR "renal replacement therapy") AND ("therapeutic drug monitoring" OR TDM OR "model-informed precision dosing" OR MIPD OR Bayesian OR pharmacometric OR pharmacometrics)	Identification of individualized dosing and monitoring approaches
PubMed	Digital and model-based dosing support	("continuous renal replacement therapy" OR CRRT OR "renal replacement therapy") AND ("machine learning" OR "digital twin" OR "Bayesian forecasting" OR "dosing software" OR "precision dosing" OR pharmacometric OR pharmacometrics)	Identification of emerging digital and model-based approaches

Source	Search domain	Representative search block	Purpose
Google Scholar	Targeted supplementary searches	Topic-specific combinations of individual drug names, RRT/CRRT, dosing, pharmacokinetics, therapeutic drug monitoring, model-informed dosing, and recently published evidence	Identification of additional relevant publications, guidelines, expert reviews, and recently published evidence not retrieved during the initial PubMed searches
Reference lists	Citation tracking	Reference lists of relevant reviews, guidelines, consensus documents, and key primary pharmacokinetic studies	Identification of additional clinically relevant or foundational publications

Note: PubMed was used as the primary bibliographic database. The PubMed search blocks shown in the table represent the structured thematic searches used to guide literature identification across predefined review domains. Searches were performed iteratively according to the topic and drug class being explored. Google Scholar and reference-list screening were used as supplementary targeted approaches and did not generate a formal screening count. The main search period was January 2015 to September 2026; older publications were retained when they provided essential physiological, pharmacokinetic, or methodological background. The last search was performed on 10 September 2026. The review focused primarily on adult populations, and pediatric-only studies were excluded. This strategy was designed for a structured non-systematic narrative review and was not intended to represent a formal systematic review strategy with duplicate screening, risk-of-bias or GRADE assessment, or PRISMA flow reporting. Broader RRT, IHD, and SLED terms were included to capture CRRT studies indexed under general renal replacement therapy terminology and to identify contextual or comparative evidence where relevant. The primary evidentiary focus of the review was CRRT in critically ill adults.

Supplementary Table S2. Published CRRT dosing regimens reported for selected anti-infective drugs

Drug	Source-reported CRRT setting	Source-reported regimen	Source-reported target / monitoring information	Source type	Main limitation	Source
Meropenem	Pre-dilution CVVH or CVVHD; effluent rate 25–35 mL/kg/h	750 mg q8h	Reported for target 40% fT > 4×MIC	Systematic review and Monte Carlo simulation	Simulation-based; applicability depends on MIC, PD target, body weight, residual renal clearance, CRRT prescription, and infusion strategy	[14]
Meropenem	CVVH; ultrafiltration rate 22 ± 12 mL/kg/h	1 g q12h for MIC ≤1 mg/L	MIC-based condition reported in source table	CRRT antimicrobial dosing review	Applies only to the reported MIC and CRRT setting; may not apply to higher MICs or higher CRRT intensity	[5]
Vancomycin	CVVH; ultrafiltration rate 30–40 mL/kg/h	400–650 mg q12h	Source text emphasizes inconsistent recommendations and individual adjustment by TDM	CRRT antimicrobial dosing review	Should not be used without concentration/AUC-guided adjustment where available	[5]
Vancomycin	CVVHD; dialysate rate 25 mL/kg/h	Loading dose 1.5 g; maintenance dose 500 mg q8h	Source table reports regimen; TDM required clinically because of high variability	CRRT antimicrobial dosing review	Fixed regimen should not replace concentration-guided adjustment	[5]

Drug	Source-reported CRRT setting	Source-reported regimen	Source-reported target / monitoring information	Source type	Main limitation	Source
Amikacin	CVVH or CVVHDF; CRRT dose 30 mL/kg/h	25 mg/kg q48h	Specific PK/PD target not reported in the summary table	CRRT antimicrobial dosing review	Aminoglycoside dosing should be individualized with peak/trough concentrations whenever available	[5]
Ciprofloxacin	CVVH; ultrafiltration rate 30 mL/kg/h	400 mg q8h	Specific PK/PD target not reported in the summary table	CRRT antimicrobial dosing review	Dose depends on MIC, infection severity, modality, and delivered flow	[5]
Fluconazole	CVVH; ultrafiltration rate 1 L/h	400 mg q24h	Specific PK/PD target not reported in the summary table	CRRT antimicrobial dosing review	Dose requirements may increase with higher ultrafiltration or dialysate rates	[5]
Acyclovir	CVVH, CVVHD, or CVVHDF; ultrafiltration and/or dialysate flow rate approximately 1 L/h	5–7.5 mg/kg q24h	Specific PK/PD target not reported in the summary table	CRRT antimicrobial dosing review	Limited evidence; neurotoxicity, renal recovery, and delivered CRRT should be considered	[5]

Note: The regimens shown are published regimens or dosing approaches reported in the cited literature for specific CRRT modalities and flow settings. They are not intended as fixed recommendations. Applicability depends on infection severity, organism susceptibility, residual kidney function, delivered CRRT intensity, membrane characteristics, treatment interruptions, non-renal clearance, body size, toxicity risk, and local TDM availability. When available, TDM or model-informed adjustment should be used to individualize subsequent dosing. Drug selection for this table should not be interpreted as prioritization or endorsement of these agents over other ICU drugs; the table is intended only to provide source-referenced examples of published CRRT dosing regimens.

Abbreviations: AUC, area under the concentration–time curve; CRRT, continuous renal replacement therapy; CVVH, continuous venovenous hemofiltration; CVVHD, continuous venovenous hemodialysis; CVVHDF, continuous venovenous hemodiafiltration; fT>MIC, percentage of time during which the free drug concentration remains above the minimum inhibitory concentration; MIC, minimum inhibitory concentration; PK/PD, pharmacokinetic/pharmacodynamic; q8h, every 8 hours; q12h, every 12 hours; q24h, every 24 hours; q48h, every 48 hours; TDM, therapeutic drug monitoring; UF, ultrafiltration.
